# Contrast Sensitivity and Stereopsis Outcomes Following LASIK Presbyopia Correction Based on the Corneal Aberration Modulation or Corneal Multifocality Induction Methods: A Systematic Review

**DOI:** 10.3390/jcm14030871

**Published:** 2025-01-28

**Authors:** Joanna Wierzbowska, Zofia Pniakowska, Anna M. Roszkowska

**Affiliations:** 1 Department of Ophthalmology, Military Institute of Medicine–National Research Institute in Warsaw, 04-141 Warsaw, Poland; 2 Optegra Eye Clinic, 02-366 Warsaw, Poland; 3 Department of Ophthalmology and Vision Rehabilitation, Medical University of Lodz, 90-549 Lodz, Poland; zofia.pniakowska@gmail.com; 4 Optegra Eye Clinic, 90-127 Lodz, Poland; 5 Ophthalmology Clinic, Department of Biomedical Sciences, University of Messina, 98182 Messina, Italy; aroszkowska@unime.it; 6 Ophthalmology Clinic, Faculty of Medicine and Health Sciences, Andrzej Frycz Modrzewski Kraków University, 31-327 Kraków, Poland

**Keywords:** presbyopia, keratomileusis, laser in situ, monovision, contrast sensitivity, stereopsis

## Abstract

**Background**. Modern laser vision correction for presbyopia treatment involves non-linear aspheric corneal ablation with the controlled induction of spherical aberration modulation to extend the depth of focus or corneal multifocality induction methods with or without micro-monovision in the non-dominant eye to provide continuous clear vision across distances. Anisometropia and the new higher-order aberrations pattern may be potential risk factors for postoperative stereopsis and contrast sensitivity (CS) deterioration. **Purpose**. The objective of this systematic review was to assess articles published until 2023 in which CS and/or stereopsis were reported following LASIK presbyopia treatment. **Methods**. We searched the PubMed, Scopus and Web of Science databases in accordance with the PRISMA 2020 flow diagram. The inclusion criteria specified original papers evaluating the outcomes of laser presbyopia correction as well as the pre- and postoperative assessment of stereopsis and/or CS. The Quality Assessment Tool was applied to assess the risk of bias. **Results**. We identified 13 studies, including 856 presbyopes (1712 eyes), with preoperative refractive errors from −11.13 D to +5.75 D, with the follow-up range between 3 and 30 months. Either contrast sensitivity improvement or no change following Presbyond^®^ Laser Blended Vision and PresbyMAX^®^ Hybrid was found in the reviewed articles. Some authors reported a significant CS reduction after symmetrical PresbyLASIK, wavefront-guided LASIK and aspheric monovision LASIK. Several studies assessing the effect of Presbyond^®^ LBV on stereopsis showed conflicting results, with the near stereopsis being reduced, unchanged or increased. A significant decrease in stereopsis was reported after aspheric monovision LASIK. **Conclusions**. The Presbyond^®^ Laser Blended Vision is a safe procedure in terms of the preservation of contrast sensitivity for presbyopia treatment. More studies are needed to elucidate the impact of aspheric corneal ablation methods or other methods inducing corneal multifocality with or without micro-monovision on stereopsis and contrast sensitivity.

## 1. Introduction

Presbyopia is a gradual, age-related loss of the eye’s ability to accommodate, which is related to the occurrence of symptoms of asthenopia at near and intermediate distances that increase over time. Presbyopia most often begins between the ages of 42 and 44 [1] and currently affects approximately 2.1 billion people in the world [2]. Presbyopia may be corrected using glasses, contact lenses, laser and surgery. Over time, the mounting demands regarding the quality of life have resulted in the increasing popularity of surgical methods for the correction of presbyopia. They include corneal procedures, phakic intraocular lens (IOL) implantation and crystalline lens extraction with multifocal IOL or extended depth-of-focus IOL (EDOF-IOL) implantation. The effective treatment of presbyopia, that eliminates or significantly reduces the dependence on reading glasses, both in emmetropic and ametropic patients, is a challenge for refractive surgeons today. Corneal approaches are widely practiced surgical procedures, as they are less invasive compared to intraocular surgery [3]. The aim of laser correction is to provide good distance, near and intermediate vision, at the same time maintaining contrast sensitivity (CS) and stereopsis, which are particularly important determinants of the quality of life [4]. A high safety profile of the procedure, quick visual rehabilitation and the stability and possible reversibility of the treatment effect are also required.

Laser in situ keratomileusis (LASIK) is the most commonly used laser method to provide functional near vision. A variety of LASIK techniques are clinically available. They include LASIK with monovision, multifocal ablation and non-linear aspheric ablation profile with micro-monovision (Presbyond^®^ Laser Blended Vision, LBV method). With the traditional monovision approach, the dominant eye has a clear focus at distance, whereas low myopia is created in the non-dominant eye to aid near vision.

PresbyLASIK is a technique whereby both eyes are adjusted for distance and near vision by creating corneal multifocality and increasing the depth of focus (DoF). In the initial PresbyMAX^®^ symmetrical procedure, a bi-aspheric profile is created in both eyes, with the central zone targeted at −1.9 diopter (D) to provide near vision, and peripheral distance zone targeted at −0.4 D. The peripheral PresbyLASIK technique is based on the creation of the distance correction at a 6-millimeter (mm) optical zone and the near correction over a 6.5 mm optical zone. This design could improve near vision both through the increased prolateness of the cornea and the DoF because of negative spherical aberrations. In currently performed PresbyLASIK modules, a component of monovision is added to multifocal corneal ablation, which provides a differential induction of DoF in dominant and non-dominant eyes (PresbyMAX^®^ µ-Monovision, PresbyMAX^®^ Hybrid) [5,6].

The Presbyond^®^ Laser Blended Vision (LBV) method combines non-linear aspheric corneal ablation with the induction of a controlled amount of spherical aberrations (SAs) in both eyes, and the creation of micro-monovision from −0.75 to −1.50 D in the non-dominant eye. The innovative, linear aspheric ablation profile takes account of the type and size of the refractive error to be corrected, the patient’s age and the preoperative SA level [7]. An SA is a naturally occurring higher-order aberration (HOA) in the human visual system that disperses the point of focus on the retina. An increased, but controlled amount of positive and negative SAs can provide a greater depth of field [8]. Toxic levels of SAs generate twilight myopia and the “halo” effect, which leads to the deterioration of the quality of vision and a reduced CS. Experimental research by Rocha et al. [9] conducted on adaptive optics simulators showed that a controlled increase in the SA level to <0.56 µm bilaterally led to the increase in the depth of corneal refractive power to 1.5 D, with the possible filtering of a blurred image by the brain after a neuroadaptation process.

All the above-mentioned laser methods of presbyopia correction based on the LASIK technique have provided a certain level of scientific evidence on clinical outcomes [10].

**Figure 1:**
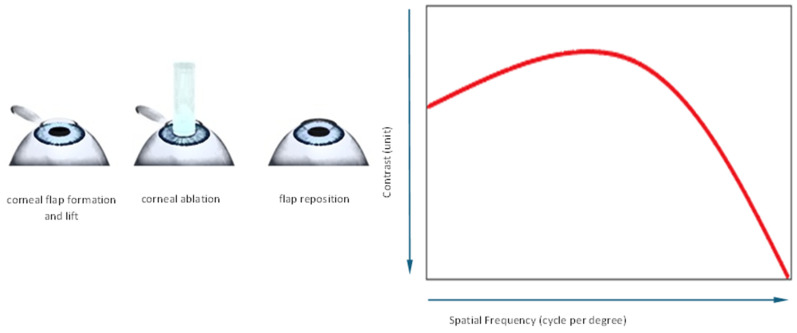
LASIK procedure and contrast sensitivity function. According to the scientific literature, LASIK procedures maintain contrast sensitivity values at the preoperative range [11].

According to the literature, standard LASIK procedures for myopia treatment do not significantly affect the contrast sensitivity function [11] (Figure 1). However, there are relatively few scientific peer-review publications on the results of testing contrast sensitivity and/or stereopsis after laser presbyopia correction with the use of modified micro-monovision LASIK procedures.

High-contrast visual acuity (VA) and contrast sensitivity measurements constitute complementary tests to visual function assessment, as full high-contrast VA does not exclude low VA with reduced contrast. CS defines the threshold between the visible and invisible and represents a person’s ability to see low-contrast images in everyday life. Stereopsis is the most sophisticated binocular function. A depth perception of less than 100 or 120 arc sec (seconds of arc) is considered to be normal stereopsis (so-called functional stereopsis), measured using either the Stereo Fly test or TNO test [12,13]. Reduced stereoacuity is associated with poorer visual functioning in everyday life and a reduction in the quality of life [4].

Some studies showed that monovision-induced anisometropia might be related to visual suppression, reduced CS and diminished binocular function [14,15]. It was shown that inducing a multifocal cornea based on the PresbyMAX^®^ or Peripheral PresbyLASIK algorithm might lead to an increase in the level of HOAs and the deterioration of CS [16,17,18]. Previously published studies discussed the refractive outcomes of the Presbyond^®^ LBV procedure in both ametropic and emmetropic eyes [7,19,20,21,22], but only a few studies presented the results of testing CS and/or stereopsis.

According to the literature, traditional monovision may be related with the impairment of stereoscopy and contrast sensitivity. Modern methods of laser presbyopia correction methods based on the control of spherical aberration (Presbyond) seem to provide better results in the context of both of the above-mentioned visual functions. This systematic review is designed to consider the impact of LASIK presbyopia treatment procedures, based on the corneal aberration modulation to extend the DoF or corneal multifocality induction or monovision methods on CS and stereopsis in patients with myopia, hyperopia and emmetropia. Our review allows the assessment of the safety and efficacy of laser presbyopia correction technologies in terms of maintaining proper stereopsis and contrast sensitivity perception in operated patients. It is the first independent summary of previous studies on this topic and includes a comprehensive collection of data from published original studies, comparing the results in tables. Our review allows for both the qualitative and quantitative assessment of the obtained results of stereopsis and contrast sensitivity after laser presbyopia correction using different LASIK techniques including Presbyond^®^ LBV, PresbyMAX^®^, PresbyLASIK, aspheric monovision LASIK and aspheric non-monovision LASIK.

## 2. Materials and Methods

The literature search was performed in accordance with the PRISMA 2020 flow diagram for new systematic reviews. Our review included the results of database and register searches regarding CS and stereopsis following laser vision correction procedures addressing the treatment of presbyopia. The PubMed, Scopus and Web of Science databases were included in our search strategy. The following terms in specific Boolean operator configuration were applied: ((presbyopia correction) OR (presbyond) OR (aspheric monovision LASIK) OR (laser blended vision) OR (presbylasik) OR (presbymax) OR (multifocal LASIK) OR (supracor) OR (monovision LASIK) AND ((stereopsis) OR (contrast)) NOT (PRK OR lens OR inlay OR pseudophakic). Only full-text, original prospective or retrospective studies published in the English language were included in this review. In addition, the main inclusion criterion was the use of either selected LASIK presbyopia correction methods based on the modulation of corneal SA to induce extended DoF or the induction of corneal multifocality with or without micro-monovision. Only studies with pre- and postoperative CS and/or stereoacuity assessment were included. Case reports, meta-analyses, review studies, conference posters and abstracts, letters to the authors, authors’ replies, unpublished studies and papers in languages other than English were excluded. Primary search results were independently assessed for eligibility by two authors, J.W. and Z.P., according to the inclusion and exclusion criteria. If disagreements occurred among the investigators, they were discussed and the senior investigator (J.W.) made the final decision.

**Figure 2:**
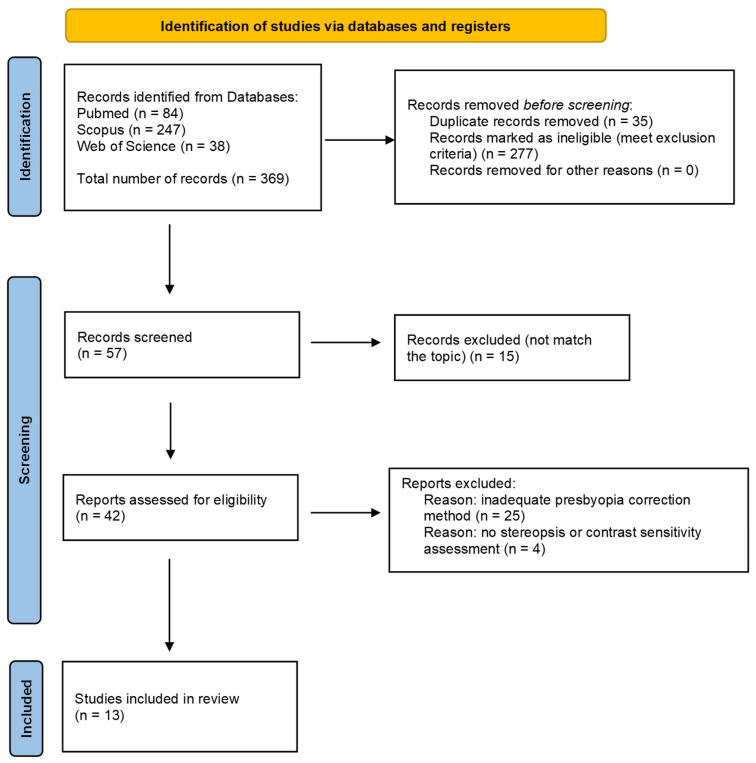
PRISMA 2020 flow diagram for the identification of studies via databases.

The literature selection for this review was presented in the PRISMA 2020 flow diagram (Figure 2). The primary number of database search results was 369 records. Subsequently, 277 records were excluded based on meeting the exclusion criteria or not meeting the inclusion criteria. Duplicates comprised 35 search results. A further screening process involved the assessment of 57 papers. Fifteen of them did not match the topic of our study. In 25 papers, an inadequate presbyopia correction was applied, and four papers lacked stereoscopy or CS assessment. Finally, 13 original studies were considered as eligible for our review of the literature. This systematic review was not registered, and the protocol was not prepared.

In the reviewed studies, presbyopia correction was performed with the following methods: Presbyond^®^ LBV [7,19,20,23,24,25,26,27,28], central multifocal LASIK (PresbyLASIK) [29], hybrid bi-aspheric multifocal central presbyLASIK (PresbyMAX^®^) [5], aspheric wavefront-guided LASIK [30] and aspheric LASIK with monovision [31].

In the Presbyond procedure designed for MEL 80^®^ or MEL 90^®^ excimer laser platforms (Carl Zeiss Meditec, Jena, Germany), the CRS-Master^®^ software platform was used to provide the program of customized non-linear ablation profiles. The profiles were based on the patients’ preoperative manifest refraction, age and the individual amount of preoperative SA. The micro-monovision targets ranged from −0.13 to −2.25 D in the non-dominant eye. Plano was applied in the dominant one.

In the central multifocal Presby LASIK procedure, the H. Eye Tech Presby-one software (Technovision GmbH, Munich, Germany) was used. The ablation produces a multifocal corneal profile at a 6 mm diameter with the central 3 mm of the treatment zone purposely rendered hyperpositively by an additional 1.50 D. A 1.5 mm circumferential transition zone of gradually changing power connects the corneal zone corrected for distance with the region corrected for near vision [29].

The hybrid bi-aspheric multifocal central PresbyLASIK procedure design includes reduced multifocality in the distant eye (target refraction +0.10 D) combined with full multifocality and micro-monovision in the near eye (target refraction −0.90 D). The software uses a treatment planning module in the aspheric mode (SCHWIND eye-tech-solutions GmbH and Co KG, Kleinostheim, Germany). Multifocality increases the range of intermediate vision with a different DoF between the distant eye (+1.10 D) and the near eye (+2.20 D) [5].

A wavefront-guided LASIK procedure was performed with the use of the VISX STAR S4^®^ excimer laser system (AMO Development LLC, Milpitas, CA, USA). WaveScan software was used to generate a custom-designed aspheric ablation profile based on the patient’s mesopic pupil size. In hyperopic presbyopes, this design steepens the central near vision zone, keeping a ratio of 1:4 of the central near vision zone and the peripheral distance vision zone, with no monovision being applied [30].

Aspheric LASIK with monovision procedure was performed with the use of the Allegretto Wave Eye-Q^®^ excimer laser (WaveLight GmbH, Erlangen, Germany). The dominant eye was corrected for far vision and the non-dominant eye was corrected for its refractive error with +1.25 D of addition for near vision. The Q-optimized F-CAT^®^ algorithm (WaveLight GmbH, Erlangen, Germany) was used to achieve the postoperative corneal asphericity of −0.80 in dominant eyes and of −1.00 in non-dominant eyes [31].

In most papers included in our review, CS was evaluated using the CSV-1000 device (VectorVision, Inc., Greenville, OH, USA) [7,19,20,23,24,25,26,27,30]. CS measurement was described elsewhere [32]. The CS scale is described with values from 0.10 to 1.35, where 1.00 represents normal contrast sensitivity. Subsequently, values above 1.00 identify supra-normal CS, while values below 1.00 indicate decreased CS when compared to the population reference ranges [24]. Other methods of CS assessment included: Contrast Glare Tester CGT-1000 (Takagi Seiko Co., Ltd., Nagano-Ken, Japan) [5], VSRC CST 1800 (Vision Science Research, San Ramo, CA, USA) [29], PR-704 Spectroradiometer (Photo Research) and Vision Works software (Vision Research Graphics) [31].

Among the vast variety of stereoscopy assessment methods, the random dot test [27], TNO stereo test (Lameris Optech BV) [24] and Titmus-C circles (Stereo Optical Co., Chicago, IL, USA) [23,28,31] were applied to measure stereoacuity following LASIK presbyopia correction in the studies included in our review. The standard random dot stereoacuity test chart for distance stereoscopy testing in 3 m includes test chart ranges from 400 to 63 arc sec. The near (40 cm) random dot tests range from 400 disparity levels of the stimuli to 12.5 arc sec. Stereopsis range, measured with the TNO stereo test for near vision, is between 480 arc sec and 15 arc sec, with lower arc sec values translating into better stereopsis quality. The near Titmus stereo circles test ranges from 800 disparity levels of the stimuli to 40 arc sec.

**Figure 3:**
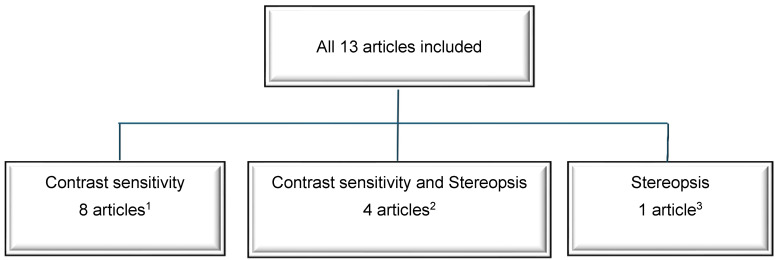
The classification of studies included in the review according to the subject of interest—contrast sensitivity, stereopsis or both parameters. ^1^ [5,7,19,20,25,26,28,29]; ^2^ = [23,24,27,31]; ^3^ = [28].

Figure 3 presents the number of included studies according to the specific subject of interest—CS, stereopsis or both parameters.

## 3. Results

Thirteen retrospective studies that reported on stereopsis and/or CS in patients treated for presbyopia were included in our review. The studies were published between 2006 and 2023. The following data were obtained from each article included in the study: the author and year of publication, mean follow-up (in months), number of patients, number of eyes, mean age of the patients (in years), sex (female/male), preoperative refractive error in D and spherical equivalent (SE), CS assessment and stereopsis assessment in arc seconds.

The studies enrolled a total of 856 participants (1712 eyes), aged from 38 to 70, with presbyopia and preoperative refractive errors that ranged from −11.13 D to +5.75 D. Seven studies assessed presbyopia treatment in both myopes and hyperopes [5,23,24,25,26,28,31], two studies assessed laser interventions in myopes [19,27], three studies in hyperopes [7,29,30] and one study in emmetropic presbyopes only [20]. Nine studies enrolled 50 or fewer patients [5,23,24,25,26,27,29,30,31], and four studies were large, including from 129 to 155 participants [7,19,20,28]. The mean observation period ranged from 3 months [27,31] through 6 [23,24,28,29] and 12 months [5,7,19,20,26,27,30], to 22 months [25]. In four papers, both stereopsis and CS were assessed [23,24,27,31]. Of the remaining nine studies, eight studies concerned CS only [5,7,19,20,25,26,29,30], and one evaluated stereopsis outcomes only [28].

In order to avoid the risk for bias, a synopsis table was created (Table 1) based on the Quality Assessment Tool for Case Series Studies from the National Heart, Lung, and Blood Institute [33]. The questions included in this tool were as follows: Q1: Is the study oriented to a clear question? Q2: Were all the patients’ results taken into account? Q3: Was the follow-up complete? Q4: Were the same conditions used in surgical treatment? Q5: Was the intervention clearly described? Q6: Was the duration of follow-up adequate? Q7: Were the results described correctly? This assessment did not determine the exclusion of any study. Articles with a high-level risk for bias had a lower weight for data synthesis.

Risk for bias assessment was classified into three evidence-level groups. Studies with zero to four “yes” answers included Romero et al. (2019) [24]. Studies with five to six “yes” answers included Reinstein et al. (2009) [7], Reinstein et al. (2011) [19], Reinstein et al. (2012) [20], Brar et al. (2021) [23], Jackson et al. (2011) [30], Alarcón et al. (2011) [31], Lim et al. (2018) [25], Reinstein et al. (2023) [26] and Zhang et al. (2016) [27]. Finally, studies with seven “yes” answers included Luger et al. (2015) [5], Alió et al. (2006) [29], Russo et al. (2022) [28].

### 3.1. Contrast Sensitivity

Twelve relevant studies which reported on CS before and after LASIK presbyopia treatment procedures were analyzed. A total of 717 participants (1434 eyes) at the mean age ranging from 38 to 68 had been followed for 3 to 30 months. Six studies assessed CS both in myopic and hyperopic presbyopes [5,23,24,25,26,31], two studies included myopic participants [19,27], three studies hyperopic [7,29,30] and one study included emmetropic presbyopes only [20]. In twelve reviewed papers, distance CS was tested [5,7,19,20,23,24,25,26,27,29,30,31]. In one study, near CS was checked [5], and in one study, CS for distance and near vision was verified [25]. The characteristics and results of the studies evaluating the impact of LASIK presbyopia treatment on CS are presented in Table 2.

The results of five studies revealed no significant changes in postoperative distance CS in comparison with preoperative outcomes in the myopic spherical equivalent group [19,27] or in groups including both myopic and hyperopic participants [23,24,25].

Four studies showed a significant improvement in CS following LASIK presbyopia treatment: in emmetropic participants [20], in a hyperopic spherical equivalent group [7] and in groups including both myopic and hyperopic participants [5,26].

In three included studies, contrast sensitivity values decreased significantly after hyperopic PresbyLASIK [29], myopic and hyperopic monovision LASIK [31] and hyperopic non-monovision LASIK [30].

Postoperative CS changes were also analyzed depending on the length of follow-up period, at 3 months, 6 months and 12 months. In the 3-month follow-up period, no statistically significant changes were found in the postoperative CS in one study [27], and a significant deterioration in contrast perception was noted in one study [31]. In the studies with a 6-month follow-up period, postoperative CS did not change compared to preoperative CS in two studies [23,24], and CS decreased in one study [29]. In the 12-month and longer follow-up period, CS did not change significantly postoperatively in two studies [19,25], improved significantly in four studies [5,7,20,26] and decreased significantly in one study [30].

In a retrospective study of 148 emmetropic presbyopes, Reinstein et al. [20] demonstrated a significant increase in postoperative mesopic CS at 3 cpd when compared to preoperative values, with no change at 6, 12 and 18 cpd. Another retrospective study conducted by this group of authors in 129 hyperopic participants revealed a significant increase in CS at 3 cpd (*p* < 0.05) and 6 cpd (*p* < 0.05)), but not at 12 cpd (*p* > 0.05) or 18 cpd (*p* > 0.05) [7]. Reinstein et al. [26] also conducted a study regarding the outcomes of Presbyond^®^ LBV in commercial and military pilots requiring class 1 medical certification. They found that the mean CS data under mesopic conditions improved significantly at all special frequencies 12 months following the surgery when compared to preoperative values. In a study by Romero et al. [24], CS increased significantly at 18 cycles per degree in the low-myopia group (<−1.00 to −3.00 D) (*p* < 0.05), and remained unchanged in the moderate myopia (from −3.0 to −6.00 D) and hyperopia groups. Lim et al. [25] demonstrated that the postoperative near CS increased significantly at 12 and 18 cpd when compared to preoperative near CS.

Similarly, Luger et al. [5] reported that 3 and 6 months after hybrid bi-aspheric multifocal central PresbyLASIK with micro-monovision, the postoperative CS log values did not differ significantly when compared to the preoperative CS outcomes. However, they improved 12 months postoperatively.

CS reduction following LASIK presbyopic procedures was reported in three studies included in this review, both in hyperopic participants [29,30] and in the myopic spherical equivalent group [31]. Alio et al. [29] reported a significant decrease in postoperative CS at 3, 6, 12 and 18 cpd (*p* < 0.05) when compared to preoperative values, with no change at 1.5 cpd (*p* > 0.05) following PresbyLASIK procedure. Similarly, Alarcon et al. [31] observed a significantly decreased binocular postoperative CS function after aspheric monovision LASIK except at the spatial frequencies of 14.8 cpd and 21.2 cpd. In the study by Jackson et al. [30], the mean CS (3 cd/m2) was significantly reduced (0.12 to 0.15 logCS per step) after aspheric non-monovision LASIK at 6, 12 and 18 cpd (*p* < 0.05) when assessed at 6 and 12 months. The reductions were, however, not clinically significant, as they were approximately 1 step (0.12 to 0.15 logCS per step) on average.

### 3.2. Stereopsis

We analyzed a total of five papers, including 284 participants (568 eyes), designed to assess the influence of LASIK presbyopia correction on stereopsis. Presbyond^®^ LBV was used in four studies [23,24,27,28], and aspheric monovision LASIK procedure was used in one study [31]. Four studies assessed stereopsis in groups that included both myopic and hyperopic presbyopes [23,24,28,31], whereas in one study, only myopic participants were tested [27]. The authors of two studies performed subgroup analysis according to the type of error [24,28] and in one study, according to the ranges of myopia: low myopia (<−1.00 to −3.00 D) and moderate myopia (from −3.0 to −6.00 D) [24]. The follow-up period ranged from 3 [27,31] to 6 months [23,24,28]. Stereopsis was tested using the following tests: the random dot test [27], Titmus test [23,28], stereo test—circles [31] and TNO stereo test [24]. Near spatial vision (40 cm) was tested in all the reviewed papers. In one study, a stereopsis test at a distance of 3 m was also performed [27]. The characteristics and results of the studies evaluating the impact of the Presbyond^®^ LBV and aspheric monovision LASIK procedures on stereopsis are presented in Table 3.

Changes in stereopsis depending on the follow-up time were summarized by dividing the included studies into two groups with the follow-up periods of 3 months and 6 or more months. In the 3-month follow-up period, a significant deterioration of stereoscopy was noted in distance stereopsis in two studies [27,31], and in one of them, there was a significant improvement in near stereopsis [27]. In the studies with a follow-up period of 6 or more months, postoperative stereoscopy did not change in one study [24] and decreased in two studies [23,28].

Zhang et al. [27] reported both a significant improvement in near stereopsis and a significant decrease in distance stereoacuity in myopic presbyopes 3 months after the Presbyond^®^ LBV procedure. Romero et al. [24] demonstrated a statistically significant improvement in stereopsis 6 months after the Presbyond^®^ LBV treatment, but only in the group of eyes with myopia from −3.0 to −6.0 D (169.4 ± 71.1 arc sec vs. 215.3 ± 99.6 arc sec; *p* < 0.05). Conversely, no changes in stereoscopic vision were noted in groups of eyes with hyperopia (183.2 ± 70.6 arc sec vs. 181.6 ± 73.8 arc sec) and with low myopia (145.7 ± 77.0 arc sec vs. 143.6 ± 80.0 arc sec). Two other authors noted a statistically significant decrease in stereopsis 6 months after the Presbyond^®^ LBV procedure [23,28]. In the study by Russo et al. [28], the average value of stereopsis was significantly compromised from 504 arc sec to 90.7 arc sec in the myopic group, and from 56.3 arc sec to 95.6 arc sec in the hyperopic group. The postoperative stereoacuity was better than 100 arc sec in 79% of myopes and 85% of hyperopes. A study conducted by Brar et al. [23] revealed a statistically significant decrease in stereopsis in the absence of correction (89.7 arc sec vs. 50.7 arc sec, *p* = 0.01). It returned to preoperative values when measured with a near addition (53.3 arc sec; *p* > 0.05).

Alarcon et al. [31] noted a significant decrease in stereopsis measured at month 3 (451.74 ± 286.97 arc sec) after the aspheric monovision LASIK procedure when compared to the preoperative values (165.55 ± 138.25 arc sec) (*p* < 0.05).

## 4. Discussion

In this review, we evaluated the effect of LASIK presbyopia correction based on corneal aberration modulation to extend the DoF or corneal multifocality induction or monovision methods on CS and stereopsis by comparing preoperative and postoperative values.

### 4.1. Contrast Sensitivity

We found that the Presbyond^®^ LBV and PresbyMAX^®^ Hybrid procedures did not compromise CS. The results of five studies revealed no significant changes in postoperative distance CS in comparison with preoperative outcomes [19,23,24,25,27]. Moreover, four other authors showed a significant improvement in CS following the Presbyond^®^ LBV treatment in myopic, hyperopic and emmetropic participants [7,20,26], or after PresbyMAX^®^ Hybrid procedure in myopic and hyperopic spherical equivalent group [5]. Some authors also reported a significant reduction in CS after PresbyLASIK, aspheric monovision LASIK and wavefront-guided non-monovision LASIK [29,30,31].

It was already shown by Campbell and Green in 1965 [34] and by Pardhan in 1990 [35] that when both eyes were best corrected, binocular CS was improved by 42% over monocular performance because of binocular summation. Conversely, binocular summation suffered as the interocular difference in retinal image quality increased [34,35]. Alarcon et al. [31] observed that although uncorrected binocular distance visual acuity was good, CS diminished significantly after monovision correction with LASIK in presbyopes. Similar findings were reported by Levinger et al. [15]. Zheleznyak et al. [36] found that binocular contrast summation was greater in the monovision that was modified by the induction of SA compared to traditional monovision.

The Presbyond^®^ LBV protocol controls the induction of positive SA in myopic eyes. In hyperopia, it induces negative SA, and in emmetropic patients it increases the number of negative SAs in the near eye while increasing the number of positive SAs in the distance eye. The association between the sign of SA and the effective DoF has been discussed. Some authors found that the induction of positive SA resulted in greater benefit for near VA compared to negative SA [7,19,36,37]. The authors of several experimental studies suggested that certain amounts of both positive and negative SA had an equal potential [38,39,40]. Bakaraju et al. [40] suggested that negative SA provided significantly larger DoF than positive SA of the same magnitude, and negative SA might be the preferred choice for near and intermediate tasks.

In the Presbyond^®^ LBV method, SA induction also improves VA with accompanying defocus in the eye with addition. Therefore, the vision of intermediate and distant objects in the non-dominant eye is much better than in traditional monovision. Other mechanisms also contribute to the high efficacy and high safety profile of this refractive procedure. They include pupil constriction during accommodation, remodeling of the corneal epithelium, the difference in the refractive index of the corneal epithelium and corneal stroma, and finally, binocular summation in the visual cortex after several months of the neuroadaptation process.

Patient selection and detailed preoperative examination, including ocular dominance assessment and “cross-blur” monovision tolerance test, are critical to successful outcomes [41]. Previous studies revealed that binocular CS was optimized when the dominant eye was assigned to distance, while ocular dominance had no significant impact on high-contrast distance VA [42,43].

In this review, we found that CS was significantly reduced at almost all spatial frequencies in hyperopic presbyopes after symmetrical central multifocal PresbyLASIK [29]. It was postulated that CS deterioration could be related to an increase in coma and decrease in SA as a result of changes in peripheral corneal asphericity following hyperopia ablation profile. We also found that the PresbyMAX^®^ hybrid method showed no change in CS at a 3- and 6-month follow-up or improvement in CS at 12 months postoperatively [5]. Previous studies on the PresbyMAX^®^ symmetrical method, with a near zone created in the center of the cornea, confirmed a postoperative reduction by at least 1 line in corrected distance visual acuity (CDVA) and corrected near visual acuity (CNVA) in 33% and 23% of patients, respectively [44]. Studies on multifocal ablation using the PresbyMAX^®^ hybrid method showed better visual outcomes than the PresbyMAX^®^ symmetrical procedure [5], which might also contribute to better CS outcomes.

This review also showed a significant CS reduction at 6 and 12 months following wavefront-guided non-monovision LASIK in hyperopic presbyopes, although the postoperative CS function still remained within the population’s normal range [30]. It was postulated by Jackson et al. [30] that a slight reduction in CS and postoperative CDVA could be associated with a relative minimization of the retinal image after the removal of corrective glasses. Conversely, comprising a patient’s individual wavefront data in the treatment design may contribute to the lower CS reduction that was observed after wavefront-guided LASIK as compared to other aspheric ablation patterns in hyperopic presbyopes [29].

### 4.2. Stereopsis

Surgically induced anisometropia may be associated with visual suppression, impaired fusion and loss of stereopsis. Alarcon et al. [31] found that stereopsis measured at 3 months after LASIK-induced monovision decreased significantly when compared to the preoperative values [31]. Wright et al. [45] confirmed an irreversible loss of stereopsis in some presbyopic patients after inducing traditional monovision using photorefractive keratectomy (PRK). The loss of stereopsis may be related to monocular suppression of the fovea in permanent monofixation syndrome and the development of a central scotoma that cannot be reversed [46,47,48]. Also, a tendency toward esophoria and decreased fusional reserves was reported after monovision treatment [49]. There is growing evidence supporting possible mediation between binocular integration and interocular suppression [50,51]. It was demonstrated by Jiang and Meng [50] that stereopsis maintenance depended on the strength of suppression. According to their findings, stereopsis could be disrupted by a large difference in an interocular image quality and intense interocular suppression, but stereopsis may survive under shallow interocular disparity and suppression.

The advantage of the Presbyond^®^ LBV method over traditional monovision is related to the use of an individualized ablation profile, taking account of the type and size of the refractive error to be corrected, the range of tolerable monovision, preoperative SA level and the patient’s age. The use of the Presbyond^®^ LBV protocol results in continuous clear vision over a range of distances, including intermediate distances. Despite the induced anisometropia, retinal correspondence and neural binocular summation are still possible, and functional stereopsis may be maintained [35]. Suppression is easier to achieve in the non-dominant eye, as the distance vision is prioritized by the patient over near vision [47].

The analyzed studies revealed some variability in stereoacuity outcomes following the Presbyond^®^ LBV procedure. Some authors reported a significant deterioration in stereopsis after the treatment [30], whereas other reports showed that patients presented comparable or significantly better stereoacuity scores when measured postoperatively [23,24,27,28]. Romero et al. [24] demonstrated a statistically significant improvement in stereopsis 6 months after the Presbyond^®^ LBV treatment, but only in the group of eyes with moderate myopia from −3.0 to −6.0 D. However, no changes in stereoscopic vision were noted in groups of eyes with hyperopia and low myopia.

Following the Presbyond^®^ LBV treatment, stereopsis may be improved by correcting refractive errors, eliminating anisometropia and eliminating the undesirable effects of glasses like minification, magnification and prismatic effects [52,53,54,55].

Several possible reasons for the unfavorable results in stereopsis in some patients following the Presbyond^®^ LBV could be considered. They include a decrease in CS, postoperative residual error, aniseikonia, anisometropia exceeding 1.50 D, increased postoperative HOAs, photic phenomena and dry eye [46,49,52,56,57,58,59].

Notably, following the Presbyond^®^ LBV treatment, Russo et al. [28] observed a decrease in stereopsis, which was minimal, and the mean postoperative stereopsis had remained within the range of functional stereopsis. The authors of the study also found that, six months after surgery, 79% of patients with myopia and 85% of those with hyperopia had functional stereopsis [28]. Reinstein et al. [60] also found that although postoperative uncorrected stereoacuity was lower than preoperative near-corrected stereoacuity after Presbyond^®^ LBV, 68% of patients had a stereoacuity of 100 arc sec or better. Conversely, Brar et al. [23] found that, 6 months after surgery, 70% of patients had a stereoacuity of 60 arc sec or better. They noted a statistically significant decrease in stereopsis in the absence of correction, which returned to preoperative values when measured with a near addition. In their study, Presbyond^®^ LBV also resulted in better reading speeds 6 months after the procedure [23].

Some authors reported a slightly higher percentage of patients with postoperative functional stereopsis among hyperopic presbyopes than among myopic presbyopes (85% vs. 79%, respectively) [28]. Several factors may contribute to such differences. Firstly, as the accommodation demand for myopes is generally lower preoperatively than in emmetropes or hyperopes owing to spectacle correction, a declined accommodation facility due to a sudden convergence demand in near tasks may be observed in myopic patients in the short-term after the treatment [61,62]. Secondly, poorer motor function of the ciliary muscle and transient decrease in near vergence amplitude may be noted in myopes [63,64]. Finally, the ablation profile for myopic eyes induces a lower shift of SA per each diopter than for hyperopic eyes, so eyes with low myopia are more challenging when reshaping the cornea with the assumed amount of SA than hyperopic eyes [28].

To the best of our knowledge, changes in stereopsis following central or peripheral PresbyLASIK and PresbyMAX^®^ have not been studied so far.

The effectiveness and safety of this refractive procedure may be achieved with careful patient selection and the preoperative exclusion of eye alignment and binocular disturbances, which may be decompensated after the surgery. Therefore, it is necessary that such parameters as phoric posture, fusional reserves and complete accommodation assessment be measured in the preoperative evaluation. Some authors recommended performing a contact lens trial to simulate the surgical outcome if any low-risk conditions were found during the evaluation [49].

### 4.3. Limitations of the Study

There were several limitations in the present study. Firstly, a limited number of articles, particularly on multifocal corneal procedures, were included in this review. Secondly, most of the analyzed studies had a retrospective design [5,19,20,23,25,26,28,31]. As was shown in a synopsis table, the follow-up was not complete in six of thirteen studies, and different conditions were used in the treatment, which might be a source of bias. Thirdly, various quantitative clinical tests were used for stereoacuity measurement in the reviewed papers, either vectographic tests or anaglyphic tests [23,24,27,40]. These tests differ in terms of the degree of dissociation (weakly dissociative tests—Stereo Fly test, Random Dot E and strongly dissociative tests—TNO test), which might yield different outcomes [65,66]. Finally, all papers included in the review had evaluated changes in stereopsis during 3-month or 6-month follow-ups only. Stereopsis may change in a long-term postoperative period during the full vision recovery process because of the plasticity of binocular vision [66]. The impact of corneal healing processes, tear film instability and the individual course of neural summation in patients in the short-term postoperative period may also be a source of outcome variability [67,68]. All above limitations restrict the ability to generalize findings of this review. However, the main strength of this study is that it is the first review to summarize the effects of different LASIK presbyopia correction methods on CS and stereopsis in a large sample of patients.

### 4.4. Future Prospects

This study identified considerable gaps within the extant literature and highlighted several areas for future investigations. Firstly, future extended follow-up studies, featuring a larger patient cohort and with strict eligibility criteria, are needed to provide more consistent evidence on the impact of different presbyLASIK procedures on CS and stereopsis. Secondly, the standardized study protocol should include the following examinations as mandatory: the defocus curve and measurement of uncorrected and distance-corrected intermediate and near visual acuity. Furthermore, the standardized evaluation of CS and the binocular vision with stereopsis, ocular dominance and the tolerance of monovision with different ranges of correction should be performed. Thirdly, high-quality prospective studies comparing different PresbyLASIK methods as well as the PresbyLASIK technique with traditional micro-monovision are needed to obtain sufficient evidence of the superiority of LASIK presbyopia correction based on the corneal aberration modulation or corneal multifocality induction methods in terms of postoperative CS and stereopsis outcomes. The study groups should be matched in terms of the type of refractive error and the level of presbyopia to diminish the risk of bias and to increase the strength of scientific evidence [10]. Finally, comparative, systematic research of the outcomes of different LASIK presbyopia techniques and the results of refractive lens exchange procedures with different types of presbyopia-correcting intraocular lenses (IOLs) would be beneficial to provide a solid evidence base for future clinical practice.

## 5. Conclusions

- Extending the DoF by inducing the controlled amount of SA in both eyes and the creation of micro-monovision to −1.50 D in the non-dominant eye is a promising binocular laser corneal approach to overcoming presbyopia.
- All studies on Presbyond^®^ LBV included in this review indicated that this aspheric micro-monovision protocol was a safe procedure in terms of the preservation of contrast sensitivity for treating myopic, emmetropic and hyperopic presbyopes.
- Three out of four analyzed studies reported an unfavorable impact of multifocal or wavefront-guided presbyopic LASIK on CS.
- In terms of the preservation of stereopsis, the results of this review are inconclusive. Several studies assessing the effect of Presbyond^®^ on stereopsis reported conflicting results, with the near stereopsis being reduced, unchanged or increased. A significant decrease in stereopsis was reported after aspheric monovision LASIK.
- Preoperative screening is of great importance when making a decision concerning laser surgery with micro-monovision. Quantitative stereopsis tests, which provide key information on the highest level of binocular function, are valuable tools to use in clinic preoperatively and after each LASIK presbyopia treatment.

## Figures and Tables

**Table 1:** Quality assessment of articles.

Author and Date	Q1	Q2	Q3	Q4	Q5	Q6	Q7
Reinstein et al. (2009) [7]	yes	yes	no	no	yes	yes	yes
Reinstein et al. (2011) [19]	yes	yes	no	no	yes	yes	yes
Reinstein et al. (2012) [20]	yes	yes	no	n/a	yes	yes	yes
Zhang et al. (2016) [27]	yes	yes	no	yes	yes	no	yes
Lim et al. (2018) [25]	yes	yes	yes	no	yes	yes	yes
Romero et al. (2019) [24]	yes	no	n/a	no	yes	yes	yes
Brar et al. (2021) [23]	yes	yes	yes	no	yes	yes	yes
Russo et al. (2022) [28]	yes	yes	yes	yes	yes	yes	yes
Reinstein et al. (2023) [26]	yes	yes	no	yes	yes	yes	yes
Luger et al. (2015) [5]	yes	yes	yes	yes	yes	yes	yes
Alió et al. (2006) [29]	yes	yes	yes	yes	yes	yes	yes
Alarcón et al. (2011) [31]	yes	yes	yes	yes	yes	no	yes
Jackson et al. (2011) [30]	yes	yes	yes	no	yes	yes	yes

n/a = not applied; Q = Question; (Q1): Is the study oriented to a clear question? (Q2): Were all the patients results taken into account? (Q3): Was the follow-up complete? (Q4): Were the same conditions used in surgical treatment? (Q5): Was the intervention clearly described? (Q6): Was the duration of follow-up adequate? (Q7): Were the results described correctly?

**Table 2:** Study characteristics—contrast sensitivity.

Study	Presbyopia Correction Method	Study Size		Female/ Male % (No. of Cases)	Age (Years) Mean ± SD or Median (Range)	SE Pre-op (D) Mean ± SD (Range)	Target in Non-Dominant Eye (D) SE (Range)	Contrast Sensitivity			Follow-Up (Months) Mean or Median
		No. of Eyes	No. of Patients					Method/ Device	Pre-op (log)	Post-op (log)	
Studies which reported no change in post-op contrast sensitivity											
D.Z. Reinstein et al., 2011 [19]	Presbyond^®^ LBV	272	136	57/43 (77/59)	median 49 (43 to 63)	Dominant eye −4.24 ± 1.69 (−0.75 to −8.38) Non-dominant eye −4.21 ± 1.64 (−1.38 to −8.38)	−1.27 ± 0.31 (−0.75 to −2.00)	CSV-1000 (VectorVision, Greenville, OH, USA)	3 cpd 0.98 6 cpd 0.96 12 cpd 0.96 18 cpd 0.93	3 cpd 0.97 6 cpd 0.96 12 cpd 0.98 18 cpd 0.95	median 12.5
D.H. Lim et al., 2018 [25]	Presbyond^®^ LBV	54	27	44/56 (12/15)	50.2 ± 7.5	−2.14 ± 2.91 (−7.50 to +3.25)	−1.44 ± 0.21 (−1.00 to −1.50)	CSV-1000E and CSV-1000- (VectorVision, Greenville, OH, USA)	Far 3 cpd 1.826 6 cpd 1.866 12 cpd 1.390 18 cpd 0.800 Near 3 cpd 1.601 6 cpd 1.567 12 cpd 1.116 18 cpd 0.337	Far 3 cpd 1.781 6 cpd 1.863 12 cpd 1.426 18 cpd 0.855 Near 3 cpd 1.649 6 cpd 1.697 12 cpd 1.313 18 cpd 0.729	mean 22.3
M. Romero et al., 2019 [24]	Presbyond^®^ LBV	100	50	n/a	46.8 ± 4.2	Group 1 +1.71 ± 0.62 (+0.50 to +3.00) Group 2 −2.11 ± 0.85 (−1.00 to −3.00) Group 3 −3.93 ± 0.87 (−3.0 to −6.00)	(−0.75 to −1.50)	CSV-1000 (VectorVision, Greenville, OH, USA)	Group 1 3 cpd 1.54 6 cpd 1.62 12 cpd 1.29 18 cpd 0.83 Group 2 3 cpd 1.47 6 cpd 1.56 12 cpd 1.34 18 cpd 1.05 Group 3 3 cpd 1.42 6 cpd 1.60 12 cpd 1.25 18 cpd 1.10	Group 1 3 cpd 1.49 6 cpd 1.56 12 cpd 1.31 18 cpd 0.84 Group 2 3 cpd 1.38 6 cpd 1.52 12 cpd 1.27 18 cpd 0.91 Group 3 3 cpd 1.49 6 cpd 1.51 12 cpd 1.18 18 cpd 0.92	mean 6
S. Brar et al., 2021 [23]	Presbyond^®^ LBV	60	30	(16/14)	50.5 ± 6.4	Hyperopic eyes +1.28 ± 1.38 Myopic eyes −2.84 ± 1.86	−1.26 ± 0.40 (−2.25 to −0.75)	CSV-1000 (VectorVision, Greenville, OH, USA)	1.5 cpd 1.5 3 cpd 1.67 6 cpd 1.58 12 cpd 1.2 18 cpd 0.72	1.5 cpd 1.48 3 cpd 1.63 6 cpd 1.54 12 cpd 1.13 18 cpd 0.63	mean 6.0
T. Zhang et al., 2016 [27]	Presbyond^®^ LBV	80	40	n/a	43.4 ± 4.9 (38 to 63)	−5.68 ± 1.98 (−1.25 to −11.13)	−1.41 ± 0.28 (−0.75 to −2.25)	CSV-1000 (VectorVision, Greenville, OH, USA)	change (*p* > 0.05) for 3cpd,6cpd, 12cpd, 18 cpd change (*p* > 0.05) for mesopic AULCSF pre-op 1.38 post-op 1.41 photopic AULCSF pre-op 1.42 post-op 1.43		mean 3
Studies which reported a significant (*p* < 0.05) increase in post-op contrast sensitivity											
D.Z. Reinstein et al., 2009 [7]	Presbyond^®^ LBV	258	129	66/34	median 56 (44 to 66)	+2.54 ± 1.16 (+0.25 to +5.75)	(−1.00 to −1.50)	CSV-1000 (VectorVision, Greenville, OH, USA)	3 cpd 0.96 6 cpd 0.94 12 cpd 0.95 18 cpd 0.90	3 cpd 0.99 6 cpd 0.96 12 cpd 0.97 18 cpd 0.92	median 12.5
D.Z. Reinstein et al., 2012 [20]	Presbyond^®^ LBV	296	148	59/41	median 55 (44 to 65)	Dominant eye +0.25 ± 0.43 (−0.88 to +1.00) Non-dominant eye +0.24 ± 0.48 (−0.88 to +1.00)	Intended: −1.52 ± 0.09 (−1.88 to −1.00) Obtained: −1.46 ± −0.42 (−2.50 to −0.38)	CSV-1000 (VectorVision, Greenville, OH, USA)	3 cpd 0.95 6 cpd 0.95 12 cpd 0.97 18 cpd 0.94	3 cpd 0.96 6 cpd 0.95 12 cpd 0.96 18 cpd 0.92	median 12.9
Studies which reported a significant (*p* < 0.05) decrease in post-op contrast sensitivity											
J.L. Alió et al., 2006 [29]	PresbyLASIK	50	25	(10/15)	58 (51 to 68)	+1.92 ± 0.68 (+0.50 to +3.50)	0	VSRC CST 1800 (Vision Science Research, San Ramon, CA, USA)	A significant reduction (*p* < 0.05) at 3, 6, 12 and 18 cpd and no change at 1.5 cpd.		6
A. Alarcon et al., 2011 [31]	aspheric monovision LASIK	50	25	n/a	49.3 ± 4.5	−1.93 ± 2.57 (+2.75 to −6.5)		PR-704 Spectroradiometer (Photo Research) and Vision Works software (Vision Research Graphics)	In dominant eyes, a significant reduction at 3 cpd (*p* < 0.05), 5.9 cpd (*p* < 0.05) and 9.9 cpd (*p* < 0.05). In non-dominant eyes, a significant reduction at all spatial frequencies, except for 18.5 cpd and 21.2 cpd. Under binocular conditions, a significant reduction at all spatial frequencies except for 14.8 cpd and 21.2 cpd.		3
W.B. Jackson et al., 2011 [30]	aspheric non- monovision LASIK	66	33	66/34	55.1 ± 4.6	+1.97 ± 0.59 (+0.75 to +3.63)	0	CSV-1000 (VectorVision, Greenville, OH, USA)	A significant reduction under mesopic conditions (3 cd/m^2^) at 6 cpd, 12 cpd and 18 cpd (*p* < 0.05).		12

No., number; SD, standard deviation; SE, spherical equivalent; D, diopter; pre-op, preoperative; post-op, postoperative; cpd, cycles per degree; arc sec, seconds of arc; n/a, not applicable; AULCSF, area under log contrast sensitivity function; deg, degree; cd/m^2^, candelas per square meter.

**Table 3:** Study characteristics—stereopsis.

Study	Presbyopia Correction Method	Study Size		Female/ Male % (No. of Cases)	Age (Years) Mean ± SD or Median (Range)	SE Preop. (D) Mean ± SD (Range)	Target in Non-Dominant Eye (D) SE (Range)	Stereopsis			Follow-Up (Months) Mean or Median
		No. of Eyes	No. of Patients					Method/ Device	Pre-op (arc sec) Mean ± SD (Range)	Post-op (arc sec) Mean ± SD (Range)	
T. Zhang et al., 2016 [27]	Presbyond^®^ LBV	80	40	n/a	43.4 ± 4.9 (38 to 63)	−5.68 ± 1.98 (−1.25 to −11.13)	−1.41 ± 0.28 (−0.75 to −2.25)	Random dot test for near (40 cm) and distance (3 m)	Near median 50 Distance median 100	Near median 45 Distance median nil	median 3
M. Romero et al., 2019 [24]	Presbyond^®^ LBV	100	50	n/a	46.84 ± 4.17	Group 1 +1.71 ± 0.62 (+0.50 to +3.00) Group 2 −2.11 ± 0.85 (−1.0 to −3.00) Group 3 −3.93 ± 0.87 (−3.0 to −6.00)	(−0.75 to −1.50)	TNO stereo test (Lameris Ootech BV)	Group 1 181.6 ± 73.8 (30 to 240) Group 2 143.6 ± 80.0 (30 to 240) Group 3 215.3 ± 99.6 (60 to 480)	Group 1 183.2 ± 70.6 (60 to 240) Gorup 2 145.7 ± 77.0 (60 to 240) Group 3 169.4 ± 71.1 (60 to 240)	mean 6
S. Brar et al., 2021 [23]	Presbyond^®^ LBV	60	30	(16)/(14)	50.47 ± 6.43	Hyperopic eyes +1.28 ± 1.38 Myopic −2.84 ± 1.86	−1.26 ± 0.40 (−2.25 to −0.75)	Titmus-C circles (Stereo Optical Co., Chicago, USA)	50.7 ± 17.2	89.7 ± 36.0	6.0 ± 1.2
A. Russo et al., 2022 [28]	Presbyond^®^ LBV	278	139	n/a	53.13 ± 5.84 (42 to 70)	Hyperopic +1.61 ± 0.98 (−1.25 to +4.63) Myopic −3.40 ± 1.83 (−0.50 to −8.25)	−0.90 ± 0.44 (−0.13 to−2.25)	Titmus Stereo Test	Hyperopic 50.5 ± 16.6 Myopic 56.3 ± 20.7	Hyperopic 90.7 ± 32.7 Myopic 95.6 ± 33.5	6
A. Alarcón et al., 2011 [31]	aspheric monovision LASIK	50	25	n/a	49.3 ± 4.5	−1.93 ± 2.57 (−6.5 to +2.75)	−1.25	Stereo Test circles (Stereo Optical Co., Inc.)	165.6 ± 138.3	451.7 ± 287.0	3

No., number; SD, standard deviation; SE, spherical equivalent; D, diopter; arc sec, seconds of arc; pre-op, preoperative; post-op, postoperative; cm, centimeter; m, meter; n/a, not applicable.

## Data Availability

The data that support the findings of this study are available upon request from the corresponding author.
